# TRPA1 modulation of spontaneous and mechanically evoked firing of spinal neurons in uninjured, osteoarthritic, and inflamed rats

**DOI:** 10.1186/1744-8069-6-14

**Published:** 2010-03-05

**Authors:** Steve McGaraughty, Katharine L Chu, Richard J Perner, Stan DiDomenico, Michael E Kort, Philip R Kym

**Affiliations:** 1 Neuroscience Research, Global Pharmaceutical Research and Development, Abbott Laboratories, Abbott Park, IL, 60064, USA

## Abstract

**Background:**

There is growing evidence supporting a role for TRPA1 receptors in the neurotransmission of peripheral mechanical stimulation. In order to enhance understanding of TRPA1 contributions to mechanotransmission, we examined the effects a selective TRPA1 receptor antagonist, A-967079, on spinal neuronal activity following peripheral mechanical stimulation in uninjured, CFA-inflamed, and osteoarthritc (OA) rats.

**Results:**

Systemic injection of A-967079 (30 μmol/kg, i.v.) decreased the responses of wide dynamic range (WDR), and nociceptive specific (NS) neurons following noxious pinch stimulation of the ipsilateral hind paw in uninjured and CFA-inflamed rats. Similarly, A-967079 reduced the responses of WDR neurons to high-intensity mechanical stimulation (300 g von Frey hair) of the knee joint in both OA and OA-sham rats. WDR neuronal responses to low-intensity mechanical stimulation (10 g von Frey hair) were also reduced by A-967079 administration to CFA-inflamed rats, but no effect was observed in uninjured rats. Additionally, the spontaneous activity of WDR neurons was decreased after A-967079 injection in CFA-inflamed rats but was unaltered in uninjured, OA, and OA-sham animals.

**Conclusions:**

Blockade of TRPA1 receptors disrupts transmission of high-intensity mechanical stimulation to the spinal cord in both uninjured and injured rats indicating that TRPA1 receptors have an important role in noxious mechanosensation in both normal and pathological conditions. TRPA1 receptors also contribute to the transmission of low-intensity mechanical stimulation, and to the modulation of spontaneous WDR firing, but only after an inflammatory injury.

## Background

The transient receptor potential (TRP)A1 receptor is part of a larger family of TRP ion channels that includes TRPV, TRPM, TRPC, TRPN, TRPP, and TRPML [1-3]. TRPA1 is expressed in small and medium-sized peptidergic primary afferent sensory neurons that also express TRPV1 [4-8]. A recent report has also demonstrated localization of TRPA1 to a subclass of large diameter primary afferent sensory neurons and epidermal kerantinocytes [8]. TRPA1 is activated by a variety of chemical irritants that include allyl isothiocyanate (mustard oil), allicin, gingerol, cinnamaldehyde, eugenol, and methysalicylate [5,6,9,10]. Local application of these TRPA1 agonists induces spontaneous pain in humans [11], as well as nocifensive behaviors and hypersensitivity to peripheral stimulation in animals [10,12].

There is now growing evidence demonstrating an important role for TRPA1 receptors in mechanotransmission [13-15]. TRPA1 ablated mice (TRPA1-/-) were less sensitive to low-intensity mechanical stimuli compared to wild-type mice, and responses to high-intensity mechanical stimulation were appreciably impaired [13]. Using the skin-nerve preparation, it was found that TRPA1 receptors in normal skin are necessary for mechanotransmission in several type of primary afferent fibers including slowly adapting C-fibers (all ranges of intensity), Aδ-fiber mechanonociceptors (only high-intensity), and slowly adapting Aβ-fibers (all ranges of intensity) [8]. Additionally, application of the TRPA1 antagonist, HC-030031 attenuated C-fiber responses to high-intensity mechanical stimulation of normal skin [16]. After an inflammatory injury, there is an increase in TRPA1 expression as well as a decrease in mechanical thresholds of TRPA1-containing primary afferent neurons [17,18]. Furthermore, mechanical sensitivity of inflamed animals was significantly decreased by systemic (HC-030031) or intraplantar (AP18) administration of TRPA1 receptor antagonists [19,20].

**Figure 1:**
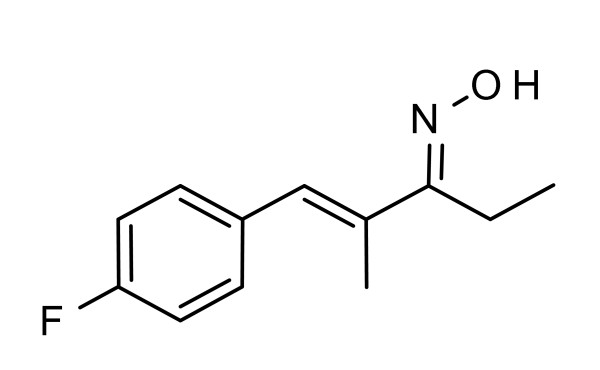
**Structure of A-967079**.

In order to further delineate the role of TRPA1 receptors in normal and pathological mechanotransmission, we administered a selective TRPA1 receptor antagonist, A-967079 (Figure 1), to examine its effects on spinal nociceptive neurons in uninjured, inflamed, and osteoarthritic (OA) rats. Systemic delivery of A-967079 has been shown to decrease mechanical sensitivity in several rat models of pathological nociception [21]. We also examined the actions of A-967079 on the spontaneous firing of spinal wide dynamic range (WDR) neurons in each of the animal models examined. Spontaneous activity of WDR neurons is heightened following a chronic injury and is an indication of central sensitization and possibly non-evoked or ongoing pain [22-27].

## Methods

All animal handling and experimental protocols were approved by Abbott's Institutional Animal Care and Use Committee (IACUC), and were conducted in accordance with the ethical principles for pain-related animal research of the American Pain Society. Male Sprague-Dawley rats (Charles River, MA, 250-400 g) were used for all experiments and were housed in a temperature controlled room with a 12/12-hr day/night cycle. Food and water were available ad libitum.

### Compound

A-967079 was synthesized at Abbott Laboratories (Abbott Park, IL). A-967079 is a TRPA1 receptor antagonist with IC_50_'s of 67 nM and 289 nM at human and rat TRPA1 receptors, respectively, and has good penetration into the CNS [21]. A-967079 (10 μM) is weak (ED_50 _> 5 μM) or not active at 89 different G-protein-coupled receptors, enzymes, transporters, and ion channels including other TRP channels (CEREP, Poitiers, France; and unpublished data), and thus is selective for TRPA1 receptors.

### Animal models

Uninjured, inflamed, osteoarthritic and sham osteoarthritic animals were used for the experiments. Osteoarthritis was induced 21 days prior to electrophysiological testing by injecting 3 mg Monoiodoacetate (MIA, 50 μl) i.a. through the infrapatella ligament of the right knee. Animals were anesthetized with 5% isoflurane followed by 2.5% maintenance during the MIA injection procedure. MIA was dissolved in 0.9% sterile saline and administered using a 29-gauge needle. Sham OA animals received an i.a injection of saline 21 days prior to testing. Chronic inflammation was induced by injecting complete Freund's adjuvant (CFA, 50%, 150 μl) into the plantar surface of the rat's right hindpaw 48 hours prior to the electrophysiology experiments.

### Electrophysiological protocol

On the day of neuronal recording, all animals were initially anesthetized with pentobarbital (50 mg/kg, i.p.). Catheters were placed into the left and right external jugular veins. A laminectomy was performed to remove vertebral segments T12-L3 for uninjured and CFA rats, and T9-L2 for OA and OA-sham rats to record from spinal neurons that receive input from either the right paw or knee, respectively. All animals were then secured in a stereotaxic apparatus (Kopf Instruments, Tujunga, CA) supported by clamps attached to the vertebral processes on either side of the exposure site. The exposed area of the spinal cord was first enveloped by agar and then filled with mineral oil. A stable plane of anesthesia was maintained throughout the experiment by a continuous infusion of propofol at a rate of 8-12 mg/kg/hr (i.v.). Body temperature was kept at approximately 37°C by placing the animals on a circulating water blanket.

Platinum-iridium microelectrodes (Frederick Haer, Brunswick, ME) were used to record extracellular activity of spinal dorsal horn neurons. The activity of WDR neurons was recorded from all groups of rats. WDR neurons were defined as those neurons that respond in a graded manner to both low- and high-intensity stimulation. The firing of nociceptive specific (NS) neurons was also recorded from CFA and uninjured rats. NS neurons were defined as those neurons that respond only to high-intensity (noxious) stimulation. Spike waveforms were monitored on an oscilloscope throughout the experiment, digitized (32 points), and then stored for off-line analysis (SciWorks, Datawave Technologies, Longmont, CO) to ensure that the unit under study was unambiguously discriminated throughout the experiment.

Each neuron was characterized to confirm WDR or NS response patterns. For CFA and uninjured animals, the neuronal receptive field (RF) on the ipsilateral hind paw was (in order) gently tapped, brushed, given a noxious pinch with forceps, and stimulated with a 10-g von Frey hair for 2-3 sec. In OA and OA-sham rats, neurons were characterized by responses to manual gentle rubbing, and a 300-g von Frey applied to the knee for 2-3 sec. Only neurons that specifically responded to knee joint stimulation, without responding to stimulation of the surrounding skin/tissue, were kept for recordings in OA and OA-sham rats.

After characterization, three baseline responses, separated by 5 min each, to specific stimulation (see below) of the neuronal RF were recorded. Spontaneous and evoked neuronal activity was then measured 5, 15, 25, and 35 min after systemic injection of A-967079 (30 μmol/kg, i.v.) or vehicle (polyethylene glycol). The intravenous injection of A-967079 or vehicle was completed over a 6-7 min period. The i.v. dose of A-967079 was selected based on extrapolated plasma levels that were effective in behavioral studies (data not shown). Except for 2 experiments in which two easily distinguished neurons were simultaneously recorded on one electrode, only one neuron was studied per rat. Since only one vehicle was used in these experiments, vehicle data was combined when a clear "no effect' was observed in at least 2-3 animals from a particular subset of groups (e.g. OA and sham OA).

Only one form of test stimulation was given in each specific experiment. In uninjured and CFA-inflamed rats, WDR responses to either low-intensity (10 g von Frey hair for 15 s) or high-intensity (noxious pinch with 22 mm mini bulldog clamp for 10 s) mechanical stimulation of the RF on the right hindpaw were recorded. Responses of NS neurons to noxious pinch (22 mm mini bulldog clamp for 10 s) of the RF were also recorded in uninjured and CFA-inflamed rats. Finally, in OA and OA-sham rats, a 300 g von Frey hair test stimulus was applied to the right knee joint for 10 s to evoke WDR activity.

### Data analysis

At the onset of each experiment, spontaneous neuronal firing was counted for 5 min to determine baseline levels (prior to characterization). Post-drug spontaneous firing was measured in the 5 min leading up to each stimulus at 5, 15, 25 and 35 min after injection. Evoked activity was measured by counting the spikes during the time of stimulus presentation. Thus, the total number of spikes was counted for 10 s during pinch and 300 g von Frey stimulation, and for 15 s during the 10 g von Frey stimulation. A mean of the three pre-drug evoked stimuli was calculated to represent baseline evoked activity. For each neuron, the post-drug spontaneous and evoked activity was calculated as a percent of the respective baseline levels.

Statistical analysis was performed on baseline levels of neuronal spontaneous activity for uninjured, CFA, OA and OA-sham rats by conducting a one-way ANOVA followed by a Fisher's LSD. Baseline levels of evoked firing for a particular stimulus were compared by using t-tests. For comparisons to baseline firing levels, statistical significance of post-drug activity was established by using a repeated-measures ANOVA followed by a Fisher's least significant difference (LSD) test. A two-way ANOVA followed by a Fisher's LSD was used for comparison of drug and vehicle groups. Post-hoc tests were only performed if the relevant parameter was significant in the ANOVA. All data are presented as mean ± SEM, and a difference was considered significant if it reached a p-value of less than 0.05.

## Results

### Baseline neuronal activity

Discharge activity was recorded from 63 WDR and 17 NS neurons from the deep dorsal horn with a mean depth of 704.7 ± 23.8 μm, and 814.7 ± 45.9 μm, respectively, from the surface of the spinal cord. Rates of spontaneous and evoked firing for WDR neurons under baseline (pre-drug) conditions in uninjured, CFA-inflamed, OA and OA-sham rats are shown in Table 1. NS neuronal activity was also examined in uninjured and CFA-inflamed rats. The mean rate of spontaneous firing for NS neurons in uninjured and CFA-inflamed rats was 0.03 ± 0.02 and 0.18 ± 0.06 spikes/sec, respectively. However, no spontaneous firing was observed in 6 of 9 NS neurons recorded from uninjured rats and in 1 of 8 neurons recorded from CFA-inflamed rats. Thus, due to the very low levels of spontaneous firing of NS neurons, this measure was not used for subsequent analysis. The mean number of baseline discharges from NS neurons following noxious pinch stimulation in uninjured and CFA rats was, 15.64 ± 5.02 and 16.42 ± 2.58 spikes/sec, respectively.

**Table 1:** Baseline discharge rates of WDR neurons

	**Spontaneous** **(spikes/sec)**	**10-g von Frey** **(spikes/sec)**	**Noxious pinch** **(spikes/sec)**	**300-g von Frey** **(spikes/sec)**
	
Uninjured	1.73 ± 0.38	10.87 ± 4.37	52.80 ± 11.34	
CFA-inflamed	4.43 ± 0.79^++^	19.99 ± 3.66*	44.19 ± 9.87	
OA	3.93 ± 0.54^+^			27.39 ± 3.83
OA-sham	2.11 ± 0.81			18.04 ± 2.45

significant vs. uninjured group ^+^p < 0.05, and ^++^p < 0.01 (Fisher's LSD), *p < 0.05 (t-test).

### Effects of A-967079 on WDR and NS neuronal activity in uninjured and CFA-inflamed rats

**Figure 2:**
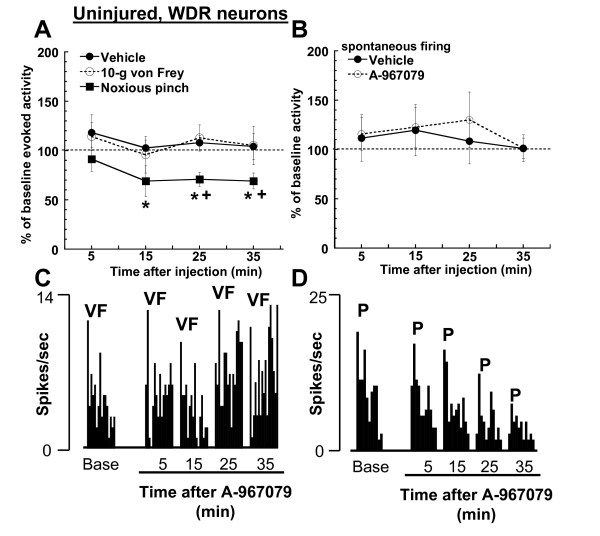
**In uninjured rats, (A) systemic administration of A-967079 (30 μmol/kg, i.v.) reduced WDR responses to noxious pinch (28 mm mini bulldog clamp for 10 sec), but not to 10-g von Frey stimulation (n = 5-7 per group) of the RF on the hind paw**. (B) Spontaneous firing of WDR neurons was unaltered by A-967079 injection to uninjured rats (n = 7-12 per group). Representative ratemeters showing the responses of a single WDR neuron to; (C) 10-g von Frey hair stimulation (15 sec), and (D) a noxious pinch (28 mm mini bulldog clamp for 10 sec) both before and after injection of A-967079 (30 μmol/kg, i.v.) to uninjured rats. VF = 10-g von Frey hair stimulation, P = noxious pinch (mini bulldog clamp). Since vehicle did not alter WDR firing in animals receiving either a von Frey (n = 5) or pinch stimulus (n = 2), this data was combined for comparison. *p < 0.05, vs. baseline firing; ^+^p < 0.05 vs. vehicle-treated group (Fisher's LSD).

Systemic administration of A-967079 (30 μmol/kg, i.v.) reduced the responses of WDR neurons to high-intensity (pinch), but not low-intensity (10 g von Frey hair) stimulation of the RF in uninjured rats (Figure 2A, C, D). The reduction in WDR responses to pinch was significant compared to both baseline levels (p = 0.02, repeated-measures ANOVA), and the vehicle group (p = 0.0002, two way ANOVA). A-967079 decreased the evoked pinch responses of WDR neurons by approximately 30% from baseline levels. This effect was observed 15 min after injection and remained at this level for the duration of recording period (35 min post-injection). Spontaneous firing of WDR neurons was not altered by the injection of A-967079 to uninjured rats (Figure 2B).

**Figure 3:**
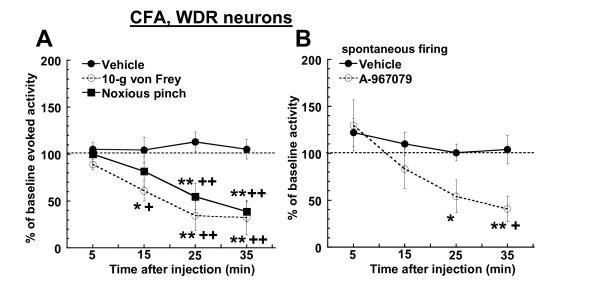
**In CFA-inflamed rats, systemic administration of A-967079 (30 μmol/kg, i.v.) reduced (A) WDR responses to noxious pinch (28 mm mini bulldog clamp for 10 sec), and 10-g von Frey stimulation (n = 6 per group) of the RF on the hind paw**. (B) Injection of A-967079 (30 μmol/kg, i.v.) also decreased the spontaneous firing of WDR neurons in CFA-inflamed rats (n = 6-12 per group). Since vehicle did not alter WDR firing in animals that received either a von Frey (n = 4) or pinch stimulus (n = 2), this data was combined for comparison. *p < 0.05, **p < 0.01 vs. baseline firing; ^++^p < 0.01 vs. vehicle-treated group (Fisher's LSD).

**Figure 4:**
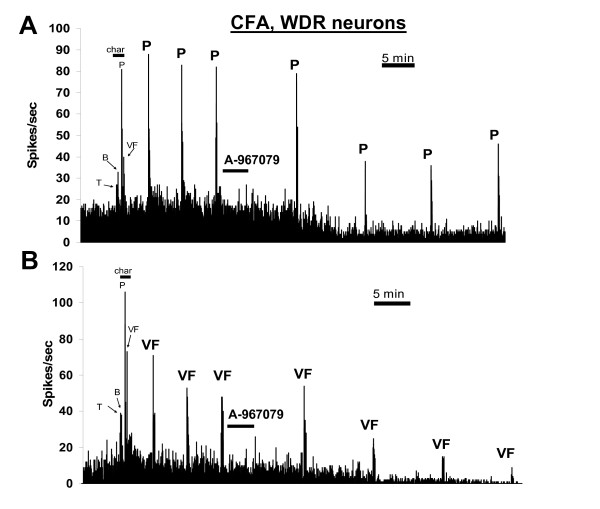
**Effects of A-967079 (30 μmol/kg, i.v.) on single WDR neurons recorded from CFA-inflamed rats**. Both evoked and spontaneous firing was attenuated by injection of A-967079. VF = 10-g von Frey hair stimulation, char = characterization period, T = non-noxious tap, B = non-noxious brush, P = noxious pinch (mini bulldog clamp). The neuronal RF on the hind paw was stimulated.

Similar to its actions in uninjured rats, administration of A-967079 (30 μmol/kg, i.v.) to CFA-inflamed rats significantly reduced WDR neuronal responses to noxious pinch stimulation (Figures 3A and 4A) compared to baseline firing (p = 0.0013, repeated-measures ANOVA) and the vehicle group (p = 0.0001, two-way ANOVA). The maximum observed effect (61.1 ± 10.97% decrease from baseline levels) on pinch-evoked activity in inflamed rats occurred 35 min after injection. In contrast to uninjured rats, injection of A-967079 to CFA-inflamed rats also significantly (p = 0.0004, and p = 0.0001 for the repeated-measures and two-way ANOVA's, respectively) reduced responses of WDR neurons to 10-g von Frey hair stimulation (Figures 3A and 4B). The maximal observed decrease in von Frey-evoked activity was 67.69 ± 18.39% from baseline levels (35 min post-injection), and was thus comparable to the effects of A-967079 on pinch-evoked activity in inflamed rats.

Also in contrast to the effects in uninjured rats, systemic injection of A-967079 significantly attenuated the spontaneous firing of WDR neurons in CFA-inflamed rats (Figures 3B and 4A, B) compared to both baseline levels (p = 0.001, repeated-measures ANOVA) and the vehicle group (p = 0.04, two-way ANOVA). This effect of A-967079 on spontaneous firing was detected by 25 min after injection, and reached a maximally observed reduction (59.27 ± 13.65% from baseline levels) 35 min post-injection.

**Figure 5:**
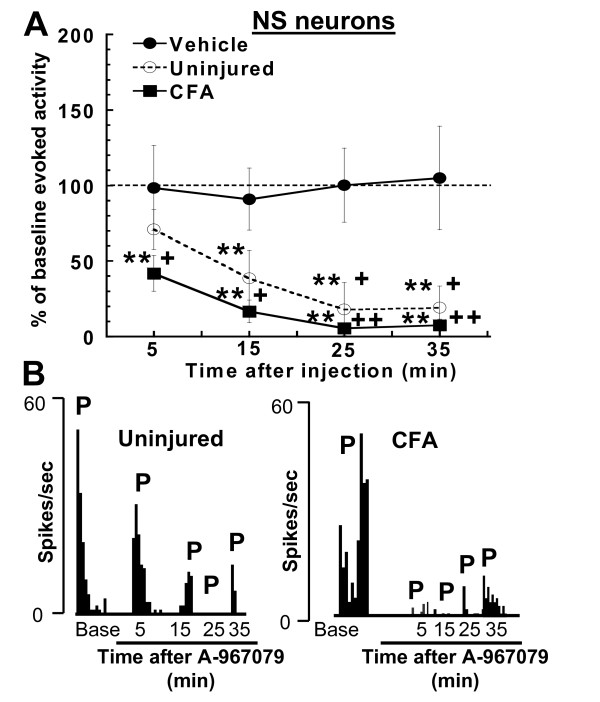
**(A) Systemic administration of A-967079 (30 μmol/kg, i.v.) reduced NS responses to a noxious pinch (28 mm mini bulldog clamp for 10 sec) in both uninjured and CFA-inflamed rats (n = 5-6 per group)**. (B) Representative ratemeters showing the responses of a single NS neuron to noxious pinch (10 sec) stimulation of the hind paw in uninjured (left), and CFA-inflamed (right) rats before and after injection of A-967079 (30 μmol/kg, i.v.). P = 10 sec pinch (mini bulldog clamp) stimulation. Since vehicle did not alter NS firing in CFA (n = 3) or uninjured animals (n = 2), this data was combined for comparison **p < 0.01 vs. baseline firing; ^+^p < 0.05, ^++^p < 0.01 vs. vehicle-treated group (Fisher's LSD).

NS neuronal activity in both uninjured and CFA-inflamed rats was substantially decreased by A-967079 (30 μmol/kg, i.v.) (Figure 5) compared to baseline levels (p = 0.0001 for both groups, repeated-measures ANOVA) as well as the vehicle group (p = 0.0004 and p = 0.0001, two-way ANOVA for uninjured and CFA rats, respectively). The pinch-evoked firing of NS neurons was significantly (p < 0.05, Fisher's LSD) reduced 5 min after injection of A-967079, and was nearly eliminated by 25 min post-injection in both groups of rats. Responses of NS neurons to noxious pinch stimulation were maximally decreased by 82.09 ± 17.55%, and 92.49 ± 3.79% from baseline levels in uninjured and inflamed rats, respectively.

### Effects of A-967079 on WDR neuronal activity in OA and sham OA rats

**Figure 6:**
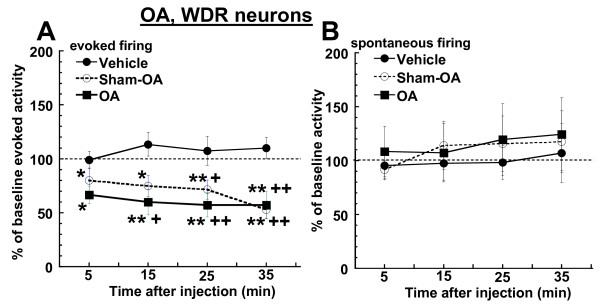
**In OA and OA-sham rats, systemic administration of A-967079 (30 μmol/kg, i.v.) reduced (A) the responses of WDR neurons high-intensity von Frey hair stimulation (300 g for 10 s) of the knee joint, but did not alter the (B) spontaneous firing of WDR neurons (n = 7-8 per group)**. Since vehicle did not alter WDR firing in OA (n = 5) or OA-sham rats (n = 2), this data was combined for comparison. *p < 0.05, **p < 0.01 vs. baseline firing; ^+^p < 0.05, ^++^p < 0.01 vs. vehicle-treated group (Fisher's LSD).

**Figure 7:**
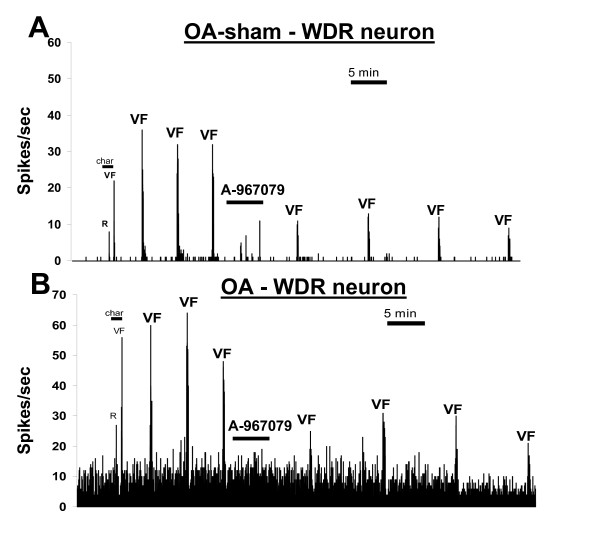
**Effects of A-967079 (30 μmol/kg, i.v.) on single WDR neurons recorded from either (A) an OA-sham or (B) an OA rat**. In both cases, A-967079 reduced the evoked responses of WDR neurons following high-intensity stimulation (300 g von Frey for 10 s), but did not change the spontaneous activity. R = gentle manual rubbing of the knee joint, VF = 300-g von Frey hair stimulation of the knee, char = characterization period.

The firing of WDR neurons was also examined in MIA-induced OA and sham OA rats. The testing parameters in these two groups of rats were limited to high-intensity stimulation due to the difficulty and questionable reliability of stimulating the knee joint through its surrounding tissue with a low-intensity stimulus. Responses of WDR neurons to a 300-g von Frey were significantly reduced from baseline levels of firing (p = 0.015 and p = 0.0009, repeated measure ANOVA, respectively) after injection of A-967079 (30 μmol/kg, i.v.) to both OA and sham OA rats (Figures 6A and 7). This evoked response was also significantly reduced compared to the vehicle group (p = 0.0001 for both groups, two-way ANOVA). The effects of A-967079 in these models were similar, as evoked firing of WDR neurons from OA rats was maximally reduced by 42.92 ± 12.3% from baseline levels, while the same stimulation was maximally decreased by 47.35 ± 8.06% in OA-sham rats at 35 min post-injection. Spontaneous firing of WDR neurons was not altered by A-967079 in either OA or OA-sham rats (Figures 6B and 7).

## Discussion

Systemic administration of the selective TRPA1 receptor antagonist, A-967079, reduced the responses of spinal WDR and NS neurons to high-intensity mechanical stimulus in both uninjured rats and in rats with OA or an inflammatory injury. These *in vivo *effects add to the growing evidence confirming that TRPA1 receptors have an important role in the transmission of noxious mechanical signals to the spinal cord under both normal and pathological conditions [8,13,16,19,20]. In contrast, the transmission of low-intensity mechanical stimulation was impeded by A-967079 only following an inflammatory injury, and was not altered in uninjured animals. Clearly, the contributions of TRPA1 to mechanosensation shift to include lower intensity stimulation following the inflammatory insult, and this data appears to be consistent with other reports demonstrating an enhanced role for TRPA1 during inflammation [17-20,28].

The neuronal responses to high-intensity peripheral stimulation were attenuated by injection of A-967079 in each of the animal models examined (uninjured, CFA-inflamed, OA, and OA-sham). The intensity of the two stimuli, a clamp-inflicted pinch and 300-g von Frey hair, would certainly be considered in the noxious range. It is likely that A-967079 acted on a similar set of pathways into the spinal cord to modulate the high-intensity stimulation across the different animal models. Furthermore, since both NS and WDR neuronal activity was modulated by A-967079, these pathways likely influence both of these classes of nociceptive neurons. Since A-967079 was administered systemically we cannot determine its exact site(s) of action, but expression of TRPA1 has been localized to different sized primary afferent sensory neurons as well as epidermal kerantinocytes [4-8]. In studies using the skin-nerve preparation, it was reported that application of a TRPA1 antagonist (HC-030031) did not alter the responses of C- and A-fibers to low-intensity mechanical stimulation of normal skin [16]. However, HC-030031 reduced the responsiveness of C-fibers once the stimulus force was increased into the high-intensity range, but Aδ-fiber activity was still unaffected. Thus, TRPA1 receptors on C nociceptors, particularly the slow-adapting subtype [13], appear to play a significant role in high-intensity mechanotransmission. Input to the spinal cord from these TRPA1-containing C-fibers was likely impeded by A-967079 administration in each of the animal models examined, and highlights the importance of this subclass of afferent neurons in transferring noxious mechanical stimulation from the periphery to the spinal cord in both normal and pathological states. It is interesting to note that while WDR neuronal responses to high-intensity stimuli were significantly attenuated by injection of A-967079, the responses of NS neurons to the same simulation were almost completely eliminated. WDR neuronal activity is influenced by a diversity of afferent fibre types, including non-TRPA1-containing fibers. Thus, it is not surprising that the responses of WDR neurons to high-intensity mechanical stimulation were not totally blocked by the injection of A-967079. When combined with the localization data, the almost complete suppression of NS neuronal firing after administration of A-967079 suggests that the mechanical responses of this neuronal class are greatly influenced by input from a specific set of C-fibers that contain TRPA1 receptors.

The transmission of low-intensity mechanical stimulation to WDR neurons was also reduced by A-967079 administration, but only in the CFA-inflamed rats. This additional action of A-967079 in CFA-injected rats indicates that TRPA1 has a role in mechanical hypersensitivity during inflammation. Consistent with this data, injection of TRPA1 receptor antagonists, HC-030031 and AP18, reduced behavioral mechanical hypersensitivity in CFA-inflamed animals [19,20]. The mechanical thresholds of TRPA1-containing primary afferent neurons have been shown to shift lower following CFA-induced inflammation [18], and may be the means through which A-967079 attenuated WDR responses to low-intensity stimulation. There are several factors that may contribute to the lowering of mechanical thresholds for TRPA1-containing neurons after CFA injection, including TRPA1 upregulation in the dorsal root ganglion [17,18], and direct activation or indirect sensitization of the receptor by bradykinin, prostaglandins, and other endogenous agents released during inflammation [8,10,28-32]. We did not examine the effects of A-967079 on low-intensity stimulation in OA rats due to the difficulty in reproducibly stimulating the knee joint through surrounding skin/tissue with a low force stimulus. Nonetheless, antagonism of TRPA1 receptors reduces mechanical allodynia in neuropathic rats [19], demonstrating that TPRPA1 receptors may have a role in pathological mechanotransmission in a model other than inflammatory injury.

A-967079 also reduced the "heightened" spontaneous firing of WDR neurons in CFA-inflamed rats. As we and others have observed previously, the spontaneous firing of WDR neurons is elevated following CFA-induced inflammation [23,24,33-35], and likely reflects injury-related sensitization of this class of spinal neurons [22-27]. Given that WDR neurons are an integral component of the ascending nociceptive pathway, and that higher levels of WDR firing generally code for increased somatosensory intensity, the observation that WDR neurons in inflamed rats spontaneously discharge at rates higher than normal may indicate that there is unevoked or "nagging" discomfort in the animal. Unevoked pain is observed in the majority of patients with chronic pain, and is a primary reason for seeking medical care [36,37]. Suzuki and Dickenson [27] have reported that heightened spontaneous discharges of WDR neurons are either reduced, or unaltered by drugs that are effective or ineffective, respectively, in treating unevoked pain in humans. Since accurate assessment of unevoked pain in animals is difficult [38], examining the ongoing firing of WDR neurons in animal models of pathological pain may provide some insight into this undesirable condition. The current data would suggest that antagonism of TRPA1 receptors may be an effective means to alleviate unevoked pain or discomfort following an inflammatory injury.

Spontaneous firing of WDR neurons was also elevated in OA rats, but unlike in CFA-inflamed rats, this increase in ongoing discharges was not altered by injection of A-967079. One difference between these models was the site of neuronal recording since input from the knee (OA) and hind paw (CFA) arrive at different sections of the lumbar spinal cord. However, both of these sections are likely influenced by fibers containing TRPA1 receptors since injection of A-967079 decreased evoked neuronal firing in both OA and CFA rats. Albeit speculative, the differential effect of A-967079 on spontaneous firing in CFA and OA rats may be more related to the degree of inflammation on the days of neuronal recording. In the MIA-induced model of OA, inflammation of the knee region has mostly dissipated by day 7 after the MIA insult [39]. Thus, inflammation would not factor into the neuronal activity recorded on day 21 in OA rats. In contrast, the release of inflammatory mediators in the CFA assay may be an important component to the augmented contributions of TRPA1 in this model [28,30,31]. These mediators probably not only factored into the effects of A-967079 on low-intensity mechanical stimulation, but also on the antagonist-related reduction of heightened spontaneous firing.

## Conclusions

The TRPA1 receptor has a significant role in the transmission of high-intensity mechanical stimulation to different classes of spinal nociceptive neurons in uninjured, inflamed and osteoarthritic animals. Additionally, there is an enhanced role for TRPA1 receptors following an inflammatory injury that includes modulation of low-intensity mechanical input to the spinal cord, and reducing elevated levels of spontaneous WDR neuronal firing. Thus, blockade of TRPA1 receptors is likely to be an effective means to alleviate pain associated with intense mechanical stimulation in both normal and pathological conditions, as well as reducing mechanical allodynia and "nagging" or unevoked pain after an inflammatory injury.

## Competing interests

The authors declare that they have no competing interests.

## Authors' contributions

SM and KLC carried out, analyzed, and designed all aspects of the electrophysiological studies. SM, KLC, and PRK were involved in constructing the original concept of the study. SM wrote the manuscript while KLC, RJP, MEC, and PRK participated in critically reviewing the manuscript. RJP, MEC, and SD were involved in the design and synthesis of A-967079. All authors have read and approved the final manuscript

## References

[B1] Clapham DE TRP channels as cellular sensors Nature 2003 426 517 524 10.1038/nature02196 14654832

[B2] Nilius B Voets T TRP channels: a TR(I)P through a world of multifunctional cation channels Pflugers Arch 2005 451 1 10 10.1007/s00424-005-1462-y 16012814

[B3] Minke B TRP channels and Ca2+ signaling Cell Calcium 2006 40 261 275 10.1016/j.ceca.2006.05.002 16806461 PMC1934411

[B4] Story GM Peier AM Reeve AJ Eid SR Mosbacher J Hricik TR Earley TJ Hergarden AC Andersson DA Hwang SW McIntyre P Jegla T Bevan S Patapoutian A ANKTM1, a TRP-like channel expressed in nociceptive neurons, is activated by cold temperatures Cell 2003 112 819 829 10.1016/S0092-8674(03)00158-2 12654248

[B5] Bautista DM Movahed P Hinman A Axelsson HE Sterner O Högestätt ED Julius D Jordt SE Zygmunt PM Pungent products from garlic activate the sensory ion channel TRPA1 Proc Natl Acad Sci USA 2005 102 12248 12252 10.1073/pnas.0505356102 16103371 PMC1189336

[B6] Kobayashi K Fukuoka T Obata K Yamanaka H Dai Y Tokunaga A Noguchi K Distinct expression of TRPM8, TRPA1, and TRPV1 mRNAs in rat primary afferent neurons with adelta/c-fibers and colocalization with trk receptors J Comp Neurol 2005 493 596 606 10.1002/cne.20794 16304633

[B7] Nagata K Duggan A Kumar G García-Añoveros J Nociceptor and hair cell transducer properties of TRPA1, a channel for pain and hearing J Neurosci 2005 25 4052 40561 10.1523/JNEUROSCI.0013-05.2005 15843607 PMC6724946

[B8] Kwan KY Glazer JM Corey DP Rice FL Stucky CL TRPA1 modulates mechanotransduction in cutaneous sensory neurons J Neurosci 2009 29 4808 4819 10.1523/JNEUROSCI.5380-08.2009 19369549 PMC2744291

[B9] Jordt SE Bautista DM Chuang HH McKemy DD Zygmunt PM Högestätt ED Meng ID Julius D Mustard oils and cannabinoids excite sensory nerve fibres through the TRP channel ANKTM1 Nature 2004 427 260 265 10.1038/nature02282 14712238

[B10] Bandell M Story GM Hwang SW Viswanath V Eid SR Petrus MJ Earley TJ Patapoutian A Noxious cold ion channel TRPA1 is activated by pungent compounds and bradykinin Neuron 2004 41 849 857 10.1016/S0896-6273(04)00150-3 15046718

[B11] Namer B Seifert F Handwerker HO Maihöfner C TRPA1 and TRPM8 activation in humans: effects of cinnamaldehyde and menthol Neuroreport 2005 16 955 999 10.1097/00001756-200506210-00015 15931068

[B12] Andrade EL Luiz AP Ferreira J Calixto JB Pronociceptive response elicited by TRPA1 receptor activation in mice Neuroscience 2008 152 511 520 10.1016/j.neuroscience.2007.12.039 18272293

[B13] Kwan KY Allchorne AJ Vollrath MA Christensen AP Zhang DS Woolf CJ Corey DP TRPA1 contributes to cold, mechanical, and chemical nociception but is not essential for hair-cell transduction Neuron 2006 50 277 289 10.1016/j.neuron.2006.03.042 16630838

[B14] Kindt KS Viswanath V Macpherson L Quast K Hu H Patapoutian A Schafer WR Caenorhabditis elegans TRPA-1 functions in mechanosensation Nat Neurosci 2007 10 568 577 10.1038/nn1886 17450139

[B15] Zhang XF Chen J Faltynek CR Moreland RB Neelands TR Transient receptor potential A1 mediates an osmotically activated ion channel Eur J Neurosci 2008 27 605 611 10.1111/j.1460-9568.2008.06030.x 18279313

[B16] Kerstein PC del Camino D Moran MM Stucky CL Pharmacological blockade of TRPA1 inhibits mechanical firing in nociceptors Mol Pain 2009 5 19 10.1186/1744-8069-5-19 19383149 PMC2681449

[B17] Obata K Katsura H Mizushima T Yamanaka H Kobayashi K Dai Y Fukuoka T Tokunaga A Tominaga M Noguchi K TRPA1 induced in sensory neurons contributes to cold hyperalgesia after inflammation and nerve injury J Clin Invest 2005 115 2393 2401 10.1172/JCI25437 16110328 PMC1187934

[B18] Dunham JP Kelly S Donaldson LF Inflammation reduces mechanical thresholds in a population of transient receptor potential channel A1-expressing nociceptors in the rat Eur J Neurosci 2008 27 3151 3160 10.1111/j.1460-9568.2008.06256.x 18598259 PMC2658012

[B19] Eid SR Crown ED Moore EL Liang HA Choong KC Dima S Henze DA Kane SA Urban MO HC-030031, a TRPA1 selective antagonist, attenuates inflammatory- and neuropathy-induced mechanical hypersensitivity Mol Pain 2008 4 48 10.1186/1744-8069-4-48 18954467 PMC2584039

[B20] Petrus M Peier AM Bandell M Hwang SW Huynh T Olney N Jegla T Patapoutian A A role of TRPA1 in mechanical hyperalgesia is revealed by pharmacological inhibition Mol Pain 2007 3 40 10.1186/1744-8069-3-40 18086313 PMC2222610

[B21] Gauvin D Mikusa J Baker S Zhong C Simler G Zhang X Bianchi B DiDomenico S Perner RJ Reilly R Kort M Jacobson P Honore P Chen J Joshi S Kym P A-967079, a potent and selective TRPA1 antagonist, is efficacious in rat preclinical pain models Society for Neuroscience Abstracts 2009 761.3

[B22] Chapman V Suzuki R Dickenson AH Electrophysiological characterization of spinal neuronal response properties in anaesthetized rats after ligation of spinal nerves L5-L6 J Physiol 1998 507 881 894 10.1111/j.1469-7793.1998.881bs.x 9508847 PMC2230815

[B23] Chu KL Faltynek CR Jarvis MF McGaraughty S Increased WDR spontaneous activity and receptive field size in rats following a neuropathic or inflammatory injury: implications for mechanical sensitivity Neurosci Lett 2004 372 123 126 10.1016/j.neulet.2004.09.025 15531101

[B24] McGaraughty S Chu KL Brown BS Zhu CZ Zhong C Joshi SK Honore P Faltynek CR Jarvis MF Contributions of central and peripheral TRPV1 receptors to mechanically evoked and spontaneous firing of spinal neurons in inflamed rats J Neurophysiol 2008 6 3158 3166 10.1152/jn.90768.2008 18829846

[B25] McGaraughty S Chu KL Scanio MJ Kort ME Faltynek CR Jarvis MF A selective Nav1.8 sodium channel blocker, A-803467, attenuates spinal neuronal activity in neuropathic rats J Pharmacol Exp Ther 2008 324 1204 1211 10.1124/jpet.107.134148 18089840

[B26] McGaraughty S Chu KL Dart MJ Yao BB Meyer MD A CB(2) receptor agonist, A-836339, modulates wide dynamic range neuronal activity in neuropathic rats: contributions of spinal and peripheral CB(2) receptors Neuroscience 2009 158 1652 1661 10.1016/j.neuroscience.2008.11.015 19063946

[B27] Suzuki R Dickenson AH Differential pharmacological modulation of the spontaneous stimulus-independent activity in the rat spinal cord following peripheral nerve injury Exp Neurol 2006 198 72 80 10.1016/j.expneurol.2005.10.032 16336968

[B28] Dai Y Wang S Tominaga M Yamamoto S Fukuoka T Higashi T Kobayashi K Obata K Yamanaka H Noguchi K Sensitization of TRPA1 by PAR2 contributes to the sensation of inflammatory pain J Clin Invest 2007 117 1979 1987 10.1172/JCI30951 17571167 PMC1888570

[B29] Brierley SM Hughes PA Page AJ Kwan KY Martin CM O'Donnell TA Cooper NJ Harrington AM Adam B Liebregts T Holtmann G Corey DP Rychkov GY Blackshaw LA The ion channel TRPA1 is required for normal mechanosensation and is modulated by algesic stimuli Gastroenterology 2009 137 2084 2095 10.1053/j.gastro.2009.07.048 19632231 PMC2789877

[B30] Cruz-Orengo L Dhaka A Heuermann RJ Young TJ Montana MC Cavanaugh EJ Kim D Story GM Cutaneous nociception evoked by 15-delta PGJ2 via activation of ion channel TRPA1 Mol Pain 2008 4 30 10.1186/1744-8069-4-30 18671867 PMC2515828

[B31] Taylor-Clark TE Undem BJ Macglashan DW Jr Ghatta S Carr MJ McAlexander MA Prostaglandin-induced activation of nociceptive neurons via direct interaction with transient receptor potential A1 (TRPA1) Mol Pharmacol 2008 73 274 281 10.1124/mol.107.040832 18000030

[B32] Materazzi S Nassini R Andrè E Campi B Amadesi S Trevisani M Bunnett NW Patacchini R Geppetti P Cox-dependent fatty acid metabolites cause pain through activation of the irritant receptor TRPA1 Natl Acad Sci USA 2008 105 12045 12050 10.1073/pnas.0802354105 PMC2575298 18687886

[B33] Peng YB Ling QD Ruda MA Kenshalo DR Electrophysiological changes in adult rat dorsal horn neurons after neonatal peripheral inflammation J Neurophysiol 2003 90 73 80 10.1152/jn.01019.2002 12634281

[B34] Kitagawa J Kanda K Sugiura M Tsuboi Y Ogawa A Shimizu K Koyama N Kamo H Watanabe T Ren K Iwata K Effect of chronic inflammation on dorsal horn nociceptive neurons in aged rats J Neurophysiol 2005 93 3594 3604 10.1152/jn.01075.2004 15659525

[B35] McGaraughty S Chu KL Faltynek CR Jarvis MF Systemic and site-specific effects of A-425619, a selective TRPV1 receptor antagonist, on wide dynamic range neurons in CFA-treated and uninjured rats J Neurophysiol 2006 95 18 25 10.1152/jn.00560.2005 16162831

[B36] Backonja MM Stacey B Neuropathic pain symptoms relative to overall pain rating J Pain 2004 5 491 497 10.1016/j.jpain.2004.09.001 15556827

[B37] Birklein F Riedl B Sieweke N Weber M Neundörfer B Neurological findings in complex regional pain syndromes--analysis of 145 cases Acta Neurol Scand 2000 101 262 269 10.1034/j.1600-0404.2000.101004262x./10770524

[B38] Mogil JS Crager SE What should we be measuring in behavioral studies of chronic pain in animals? Pain 2004 112 12 15 10.1016/j.pain.2004.09.028 15494180

[B39] Bove SE Calcaterra SL Brooker RM Huber CM Guzman RE Juneau PL Schrier DJ Kilgore KS Weight bearing as a measure of disease progression and efficacy of anti-inflammatory compounds in a model of monosodium iodoacetate-induced osteoarthritis Osteoarthritis Cartilage 2003 11 821 830 10.1016/S1063-4584(03)00163-8 14609535

